# Developing an Automated Virtual Reality Therapy for Improving Positive Self-Beliefs and Psychological Well-Being (Phoenix VR Self-Confidence Therapy): Tutorial

**DOI:** 10.2196/51512

**Published:** 2024-08-07

**Authors:** Laina Rosebrock, Jason Freeman, Aitor Rovira, Andre Lages Miguel, Rupert Ward, Matthew Bousfield, Ludovic Riffiod, Roya Kamvar, Thomas Kabir, Felicity Waite, Daniel Freeman

**Affiliations:** 1 Department of Experimental Psychology, University of Oxford, Oxford, United Kingdom; 2 Oxford Health NHS Foundation Trust, Oxford, United Kingdom; 3 The McPin Foundation, London, United Kingdom

**Keywords:** virtual reality, VR, psychosis, cognitive behavioural therapy, psychological well-being, design process, self-beliefs, psychological therapy, real-world, efficacy, well-being, mental health, participant, stakeholder, user, Phoenix VR Self-Confidence Therapy

## Abstract

Virtual reality (VR) is an immersive technology in which delivery of psychological therapy techniques can be automated. Techniques can be implemented similarly to real-world delivery or in ways that are not possible in the real world to enhance efficacy. The potential is for greater access for patients to effective therapy. Despite an increase in the use of VR for mental health, there are few descriptions of how to build and design automated VR therapies. We describe the development of Phoenix VR Self-Confidence Therapy, designed to increase positive self-beliefs in young patients diagnosed with psychosis in order to improve psychological well-being. A double-diamond, user-centered design process conducted over the course of 18 months was used, involving stakeholders from multiple areas: individuals with lived experience of psychosis, clinical psychologists, treatment designers, and VR software developers. Thirteen meetings were held with young patients diagnosed with psychosis to increase the understanding and improve the assessment of positive self-beliefs, help design the scenarios for implementing therapeutic techniques, and conduct user testing. The resulting Phoenix therapy is a class I United Kingdom Conformity Assessed (UKCA)–certified medical device designed to be used on the standalone Meta Quest 2 (Meta Platforms) headset. Phoenix aims to build up 3 types of positive self-beliefs that are connected to psychological well-being. In a community farm area, tasks are designed to increase a sense of mastery and achievement (“I can make a difference”); in a TV studio, users complete an activity with graded levels of difficulty to promote success in the face of a challenge (“I can do this”); and in a forest by a lake, activities are designed to encourage feelings of pleasure and enjoyment (“I can enjoy things”). Phoenix is delivered over the course of approximately 6 weekly sessions supported by a mental health provider. Patients can take the headsets home to use in between sessions. Usability testing with individuals with lived experience of psychosis, as well as patients in the National Health Service (aged 16‐26 years), demonstrated that Phoenix is engaging, easy to use, and has high levels of satisfaction.

## Introduction

Virtual reality (VR) has the potential to automate the delivery of powerful psychological therapy techniques. This includes direct translation of evidence-based techniques that are used in traditional face-to-face therapies as well as techniques that are not possible or difficult in the real world, potentially enhancing efficacy. The automation of such techniques means therapies can be more widely available in clinical services. However, automation of psychological therapy in VR requires careful execution to ensure there is precise translation of effective techniques that link to clear clinical targets. In order for an automated VR therapy to be effective, the design process must be methodically and rigorously conducted.

We previously published 2 papers on the design process of gameChange [1,2], a VR therapy targeting agoraphobic avoidance in patients with psychosis. Most VR therapies have been used to target avoidance of feared situations and reduce anxiety [3]. We describe in this paper the development of a new VR therapy with a different clinical target: the improvement of positive self-beliefs and psychological well-being. Phoenix VR Self-Confidence Therapy uniquely combines techniques derived from existing therapies with techniques that differ from those used in the real world to target key processes that impact psychological well-being. Additionally, since the development of gameChange, there have been improvements in VR hardware and programming capabilities. Primarily, this has included the use of standalone headsets (ie, a gaming laptop is not required to run the program, nor external cameras) such as the Meta Quest 2 (Meta Platforms). Standalone headsets are more affordable and accessible for patients and do not require a staff member to set them up and be present throughout use.

## VR for Psychosis

VR allows for the creation of 3D computer simulations of environments that can be interacted with in a similar way to the real world. Virtual environments elicit similar responses to their real-world counterparts [4]. This makes it possible for people to enter environments in the virtual world and learn new things that can then apply to real-world situations. VR has a number of advantages. It allows for full control over the environment, including specific stimuli. Scenarios can be repeated and entered gradually. People are more willing to enter virtual scenarios precisely because they know the situations are not real; indeed, one study indicated that three-quarters of people would rather participate in VR-based exposures than real-world exposures [5]. Lastly, VR therapies can be highly engaging; therefore, they are more appealing for patients [6].

The use of VR among patients with psychosis has included aided therapies (ie, using VR alongside traditional face-to-face therapy). For example, Pot-Kolder and colleagues [7] compared participants who received VR-based cognitive behavioral therapy (VR-CBT) to those in a wait-list control group and found that those who received VR-CBT had reductions in paranoid ideation and anxiety. Patients received 16 sessions with a psychologist, and VR was used together with face-to-face therapy: psychologists provided in-the-moment feedback and guidance on how to respond to situations differently while the patient was in the headset. Another study examined the initial feasibility and efficacy of VR therapy to target the impact of negative symptoms (eg, poor motivation, blunted affect, and difficulties experiencing pleasure). Patients received 12 sessions of VR therapy using principles based on existing evidence-based therapies (CBT and cognitive remediation). Environments were simulated real-world scenes (eg a factory, pub, and rooms that may be found in one’s house, such as a TV room). The VR therapy was found to be acceptable and engaging, with patients indicating increased levels of goal attainment [8]. As with the Pot-Kolder et al [7] study, all sessions were completed with a graduate-level psychologist, and tasks in VR were discussed directly with the therapist in each session.

A significant advantage of VR is that therapy can be automated and psychological principles embedded within the VR program, including through the use of a virtual coach. This allows the therapy to be supported by a range of staff members, including peer support workers and graduate mental health workers, thereby making it accessible to many more people. An automated VR therapy developed by our team was shown to significantly reduce fear of heights over the course of 2 weeks [9]. In the largest trial of VR with patients to date, an automated treatment developed by our team (gameChange) was found to be effective at reducing agoraphobic avoidance in patients with psychosis, particularly for people with severe levels of avoidance [10,11]. VR scenarios were simulated everyday situations, such as a physician’s clinic, bus, and café. Both gameChange and Fear of Heights include a virtual coach who describes the treatment principles and guides patients through the program. Another study conducted by our team compared an automated VR cognitive intervention with automated VR mental relaxation to reduce paranoia and persecutory beliefs in patients with psychosis. Both therapies included 4 sessions including anxiety reduction techniques. The cognitive intervention was delivered directly in VR by a virtual coach while the mental relaxation intervention included audio files of relaxation techniques. Both interventions were supported by a clinical or assistant psychologist and produced large reductions in paranoia [12].

The studies mentioned above all use exposure-based techniques to target anxiety, social avoidance, and paranoia, which are typical treatment targets in traditional face-to-face therapies for psychosis. They focus on encouraging patients to enter simulations of real-world scenes such as a café, shopping center, or pub in order to learn that they are safe and capable of coping with anxiety. However, there are other important treatment targets for patients with psychosis that may benefit from interventions that encourage users to enter situations they may not encounter in everyday life.

## Positive Self-Beliefs and Psychological Well-Being

Our new treatment focused on a different clinical issue in psychosis: low self-confidence and psychological well-being. Patients with psychosis can be self-critical, see themselves as inferior to others, and feel a sense of failure [13]. Psychological well-being is often low [14]. Negative self-beliefs exacerbate the experience of other psychotic symptoms, including paranoia, voice-hearing, and lack of motivation. The result is often a withdrawal from everyday activities. Withdrawal further increases the sense of defeat and means there are fewer opportunities for people to have positive experiences, thereby lowering self-confidence even more. A survey of 1800 patients with schizophrenia found that increasing self-confidence is 1 of the top 3 treatment goals [14].

Psychosis often first presents in late adolescence or the early twenties [15,16]. This is an age at which many people are in school or university, beginning employment, and experiencing other significant life events (eg, moving out of the family home, entering into a significant romantic relationship, and making new friends). Such changes can affect one’s self-beliefs and psychological well-being and have a lasting impact. Therefore, this is a key time to intervene as there are greater connections on which to build (ie, colleagues and friends, hobbies and interests, a sense of purpose through employment or study, and family relationships). Indeed, intervening early during the experience of psychosis has been shown to improve clinical outcomes [17,18].

Effective face-to-face interventions, drawing on CBT and positive psychology techniques, can improve self-confidence [19-22]. Some of these techniques involve helping patients learn to increase positive self-beliefs—a mechanism for improving psychological well-being—through direct experience (ie, being in situations in which these positive self-beliefs can be activated and maintained). It is these techniques that lend themselves particularly well to implementation in VR therapies.

## Design Process

### Overview

An automated VR program with embedded therapeutic principles requires a design process that ensures these principles are correctly targeted and implemented. This means input from end users is imperative. The overall aim is to create a program that is easy to use, meaningful, accessible, and highly satisfactory. The design process for Phoenix specifically used a double diamond approach to achieve this aim. Similar to gameChange [1], we used a person-centered design process within the double diamond approach. This is an iterative approach that ensures that the end user’s perspective and needs are incorporated into all stages (in this case, the end users are patients with psychosis).

The double-diamond design approach, developed by the Design Council [23], involves exploring an issue widely then taking focused action. It is organized into 4 phases: discover (understanding lived experience of the clinical problem and enhancing understanding and assessment of the key target), define (set out the design brief), develop (refine storyboards, program the application, and perform user testing), and deliver (finalize implementation, perform debugging, and conduct final usability testing). The design process for Phoenix will therefore be described within each of these phases. The double diamond approach was used for the overall look, feel, and design of Phoenix, as well as for individual elements within the program itself (eg, the virtual coach). Prior to beginning the design process, a young person’s advisory group (YPAG) was formed, facilitated by the McPin Foundation (a foundation that aims to include people with lived experience of mental health difficulties in mental health research). The YPAG included 14 members aged 16‐30 years with lived experience of the clinical problem (ie, low levels of positive self-beliefs in the context of the experience of psychosis).

The research team at Oxford included clinical psychologists, a treatment designer, and the software development team (3 VR programmers, a 3D artist, and a lead computer scientist). As with gameChange, the overall responsibility for final design decisions was held by the chief investigator at Oxford.

### Discover

Before the first meetings with the YPAG, clinical psychologists and the treatment designer met weekly over the course of 2 months (January and February 2021) to develop a basic design brief that would form the basis for initial discussions with the YPAG. This design brief set the boundaries for the treatment and how techniques could be implemented in VR. The initial aim of the discover phase was therefore to further elaborate this design brief. Using clinical expertise and findings from previous face-to-face studies [19,24], we set the key mechanisms to be targeted as well as the psychological techniques used to target these mechanisms. The resulting design brief outlined the clinical problem (low self confidence and psychological well-being), the target audience (young people with a diagnosis of psychosis), the clinical target (positive self-beliefs), the key function of VR (provide experiences of positive beliefs in VR to stimulate engagement in real-world activities), essential elements (eg, a virtual coach who would guide users through the program), and how it might fit in existing services (a discrete form of standalone therapy that patients could take home and use with the support of a mental health provider and supplementary materials). The script for the program needed to effectively communicate psychological principles using accessible language and ensure that the content was appropriate for the users. Additionally, the program needed to be certified as a medical device and therefore developed with regulatory and safety procedures in mind.

The first meetings with the YPAG primarily centered around clarifying the terminology used to describe self-confidence, as well as specific activities and situations in everyday life that increase self-confidence and psychological well-being. At least one member of the software development team, as well as all members of the research team, attended these meetings. Given the clinical target (positive self-beliefs), an important aspect of these initial meetings with the YPAG therefore included the identification of specific positive self-beliefs associated with increased self-confidence and psychological well-being. It became clear that existing measures did not provide a wide enough range of positive self-beliefs, did not use clear and precise wording, and would not select those in greatest need of interventions to improve such beliefs. It was agreed that a well-validated measure of positive self-beliefs—informed by people with lived experience—should include key beliefs highly connected to psychological well-being that can be targeted by psychological approaches.

Therefore, as part of this discovery phase, we developed such a measure: the Oxford Positive Self Scale (OxPos) [25]. Through discussions with the YPAG, it was agreed this measure should assess the key types of positive self-beliefs to be targeted in VR. Three key types were identified: those relating to mastery and achievement (“I have a purpose,” “I am capable,” and “I can achieve things”); those relating to strength and resilience in the face of a challenge (“I am strong,” “I am resilient,” and “I don’t give up”), and those relating to enjoyment (“I can relax,” “I can have fun,” and “I can switch off”). These beliefs fed into all aspects of programming. The validation study for the OxPos began during this phase and resulted in a 24-item, easy to use, and psychometrically robust assessment of positive self-beliefs linked to psychological well-being.

The YPAG also discussed their views on the virtual coach who would guide users through Phoenix. Different ideas about the coach were shared, including the possibility of a coach that was nonhuman (ie, a robot or creature). Ultimately, it was agreed that a human coach that was friendly, kind, and was ethnically ambiguous and relatively gender neutral in appearance would be maximally relatable and therefore most effective for guiding patients through Phoenix. Emotional attributes such as the warmth of facial expressions and animations such as head nodding were highlighted as particularly important for therapeutic alliance [26]. Furthermore, following discussions on the importance of terminology used to describe self-confidence and positive self-beliefs, initial ideas on the script for the coach were developed during meetings among the research team. During this phase, how the coach would describe the rationale behind Phoenix was discussed: that often, feelings of vulnerability and difficult experiences can allow negative self-beliefs to flourish and make it difficult to access positive self-beliefs. Therefore, Phoenix is about redressing this balance.

Given that any suggestions for activities and environments in VR needed to be programmable, weekly meetings among the clinical psychologists, treatment designer, and software development team at the University of Oxford were held in parallel to meetings with the YPAG. Additionally, at least one member of the software development team attended meetings with the YPAG to provide input on what was programmable. Due to the COVID-19 pandemic, meetings were conducted remotely over Zoom (Zoom Video Communications Inc).

### Define

The design brief was used as a living document that, in the define phase, was elaborated in greater detail to set the framework for the software development team. This included identifying the broad virtual environments linked to the 3 key types of positive self-beliefs and the activities within these environments. For mastery and achievement, YPAG members particularly identified activities that would tap into a sense of responsibility, such as caring for animals. They also highlighted the importance of simply completing tasks (ie, via a to-do list). For strength and resilience, it was agreed that patients should enter a challenging situation that would allow them to learn that they could complete a task despite feeling anxious and therefore feel a sense of accomplishment. Finally, for enjoyment, members described wanting a situation that was relaxing, where patients could immerse themselves in their surroundings and enjoy the experience in the moment. It was agreed that this situation should include an outdoor space.

Three environments were chosen: a community farm for mastery and achievement, a TV studio for strength and succeeding in the face of a challenge, and a forest by a lake for pleasure and enjoyment. Initial sketches were drawn by the 3D artist and provided to YPAG members in both 2D and 3D format for feedback. When discussing activities to include in each environment, the importance of multiple types of positive feedback was emphasized (eg, animals’ facial expressions or behavior, comments from the coach, and positive sounds when tasks were completed). Therefore, these were incorporated in all activities. In addition to the 3 environments where users would complete activities, a fourth area was highlighted as important: the welcome room where users were initially greeted by the virtual coach. It was decided that this room should be a calming, indoor space with elements of nature (ie, plants, a room overlooking mountains). Similar to the other environments, sketches were drawn by the 3D artist and provided to the YPAG members for feedback. We also discussed having uplifting music playing in the background; the music chosen was also provided to the YPAG for feedback.

In this phase, the features and appearance of the coach were also finalized with feedback provided by the YPAG on the look and feel (ie, hair color, body shape, and clothing) as well as the voice (eg, male vs female and young vs old). The YPAG indicated that they would prefer a female coach. They wanted a body shape that was tending more to gender neutral (eg, not too feminine or masculine) and average in terms of size and height (eg, not too thin). Her name (Farah) and voice were therefore also finalized. Several voice actors were identified from the Royal Academy of Dramatic Art in London, United Kingdom, and clips were sent to the YPAG for feedback. A voice actor was chosen and recording sessions for the script took place in later phases of the design process. This occurred concurrently with the development of the script.

The 3D artist also created an overall visual design manual detailing the general look and feel of each element of the program (including textures, lighting, color, animation, and stylization) to ensure consistency across the environments. The name of the therapy (Phoenix) and logo were also designed during this phase, and feedback was provided by the YPAG. Phoenix was suggested by the research team given that, in Greek mythology, a phoenix is associated with the sun and a sense of renewal. This fitted with the aim of the therapy—to encourage patients to build up positive self-beliefs and rediscover themselves. The logo was drawn by a consultant graphic designer and included suggestions of color schemes, images of the phoenix bird, and different fonts for the name.

Altogether, across both the discover and define phases, 7 virtual meetings were held with the YPAG over the course of 3 months (February-May 2021), totaling 76.5 YPAG person-hours. Email consultations on the final version of the OxPos questionnaire, logo, and project and coach names totaled an additional 19.75 YPAG person-hours.

### Develop

#### Overview

**Figure 1.**
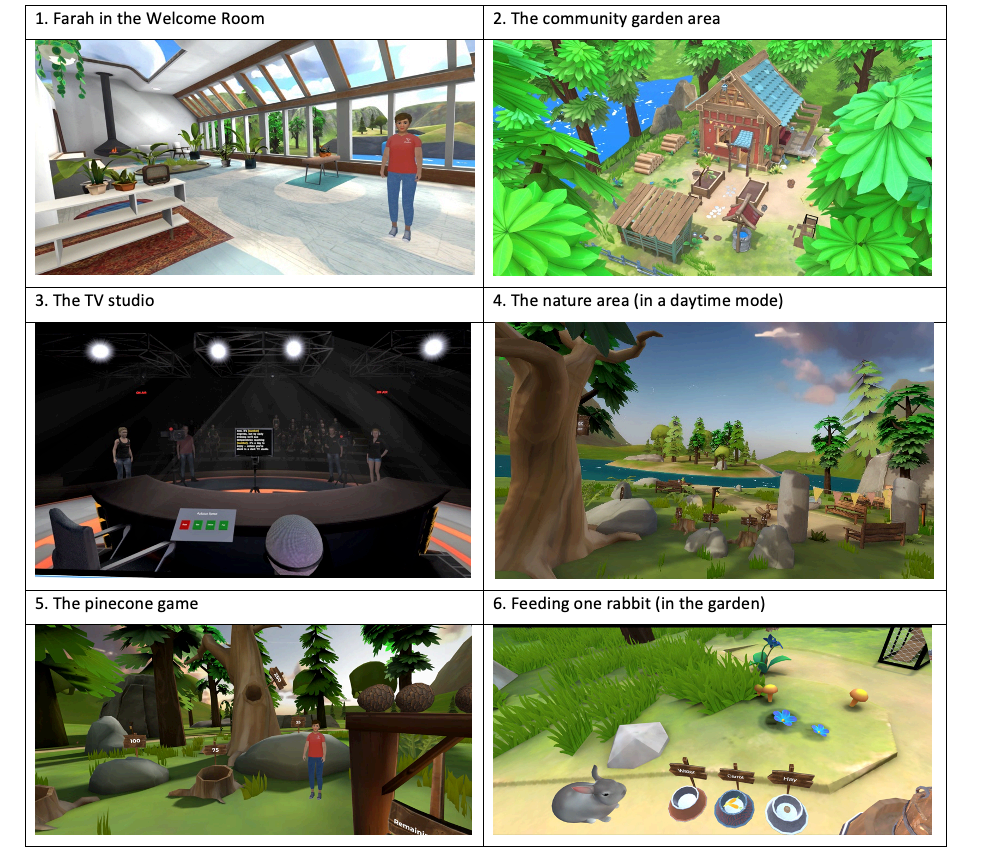
Pictures of the areas within the Phoenix VR Self-Confidence Therapy program.

The develop phase (June 2021-August 2022) included finalizing and programming the specific activities within each of the 3 main environments, as well as the welcome room (Figure 1 provides screenshots). The welcome room includes large glass-fronted windows looking out onto waterfalls, a lake, and mountains, with the room itself containing plants and a fireplace. Users can hear the sounds of birds and water splashing and uplifting music. The coach greets users in front of the window while describing the purpose of Phoenix and providing a tutorial on how to use the equipment to navigate through the program.

In the mastery and achievement scenario (the community farm), 3 key activities were chosen, comprising 10 steps: caring for animals (feeding one animal, building a hutch, and then feeding multiple animals), growing plants (preparing the bed, planting the seeds, watering the seeds, and picking crops), and redecorating a house (hanging and populating items on shelves, hanging pictures on the wall, and building a bird feeder). Rabbits were chosen as the animals due to patient preference and design complexity (ie, rabbits are relatively easy to design, animate, and program in VR compared to other animals).

In the scenario designed to promote success in the face of a challenge (the TV studio), the key element was being the center of attention and having to perform a specific task, where an audience would gradually increase in size and provide positive feedback. The resulting activity requires users to read out preexisting text from a teleprompter, with the scene being gradually populated by an increasing number of characters (eg, crew and audience). The audience (when present) claps at the end of each reading. Other elements to increase difficulty include the addition of cameras (being both off and on), spotlights, and “on air” signs. The resulting scenario includes 10 levels of difficulty, with the final 2 levels requiring users to make up a weather report rather than read a preexisting one from the teleprompter.

For the pleasurable scenario (the forest), the main programming focus was to ensure the scene was rich in terms of audio (eg, birds singing, owls hooting, water splashing) and visual detail in order to ensure full immersion. Four activities were ultimately chosen: 2 exercises to promote relaxation (a savoring exercise, whereby users are encouraged to focus on different elements of the environment, and a progressive muscle relaxation exercise, both delivered by the coach) and 2 games purely for fun (a game whereby users throw pinecones into holes in trees for points and a drumming game with different modes). The YPAG also suggested giving users choice about whether they complete activities in daylight or at night—therefore, this is built into each exercise and each mode has different sounds and sights.

The script for the dialogue of the virtual coach evolved as activities were programmed. Preferences for wording were elicited through feedback from the YPAG at all stages of the design process. As the scenarios in Phoenix do not include social interaction with other virtual characters, the script only includes lines delivered by the virtual coach. Initial voice recording for the script took place at a recording studio in Oxford over 2 sessions in June and August 2022.

#### Software Development

The development of the Phoenix software was carried out by the software development team (3 VR programmers, a 3D artist, and a lead computer scientist) within the research team at the University of Oxford. A key requirement was for Phoenix to run on a standalone headset to allow for greater accessibility and portability (ie, ensuring that participants could take the headsets home and run Phoenix themselves, without the need for additional hardware such as a laptop and link cable). Therefore, Phoenix was developed to run on a Meta Quest 2 headset.

The 3D model for the virtual coach was created by the 3D artist using Character Creator (Reallusion)and ZBrush (Maxon). This model was then animated using iClone (Reallusion) software, including blinking and lip-sync. The coach was programmed to look at the user when speaking to them (or at other objects in the scene at relevant moments). Virtual environments and interactive objects were modeled using 3ds Max and Maya (both Autodesk) and textured using Substance Painter (Adobe).

The 3D models were imported into a project in the Unity game engine (Unity) where they were assembled into individual scenes. The programmers added behaviors to create the various activities and other scripts to control positioning and navigation. The player rig and object interactions were based on Oculus Unity Integration (Meta Platforms), with enhancements provided by HurricaneVR (CloudWalkin Games).

A database was created in the cloud using Azure (Microsoft) to facilitate identification of users, store their progress, and receive a log of each session generated in real time by the application. The session logs contained a chronological list of details of significant events occurring during the session to facilitate future analyses. This system allowed for offline use, with data generated while the headset was not connected to the internet being stored on the headset so that it could be synced with the server later when an internet connection was available.

For efficiency of development, an iterative approach was used whereby initial prototypes of environments and activities were checked by the research team at the University of Oxford for usability and effectiveness. Thus, the initial design and script were continuously honed as each build of the application was distributed and reviewed. Some decisions regarding scoping and descoping of design elements became clearer only once a prototype containing the whole experience in some form was available. Once elements and activities were more fully programmed, we ran usability testing sessions with members of the YPAG.

#### Usability Testing

Usability sessions were completed in person with members of the YPAG as well as 2 members of another project’s patient involvement group to ensure a diversity of users. Testing sessions took place at the Warneford Hospital site at the University of Oxford in Oxford, United Kingdom, and the McPin Foundation offices in London, United Kingdom. At least one member of the research team (clinical psychologist or treatment designer) and one member of the software development team were present for all sessions. Sessions lasted approximately 2 hours, with the first 1‐1.5 hours spent using Phoenix (with breaks) and approximately 30 minutes for debriefing. A usability questionnaire, created by our team, was given to all users during the final user testing session. Users were offered the choice to complete the questionnaire on their own or with help from the research team. Additional feedback was elicited during the debrief using specific questions about what they had done in VR (ie, how they found interacting with the objects, what they thought of the activities, and whether the coach was helpful).

The first usability session was held with 4 users in May 2022 to provide feedback on the farm activities, welcome room, TV environment, and forest environment (particularly the pine cone game). The second usability session was held with one user in June 2022 and the third and final usability session was held in August 2022 with 7 users (3 of whom had attended the earlier user testing sessions and 4 of whom had not) to review all activities and provide feedback on Farah, the virtual coach. At this point (August 2022), only 50% of the coach’s lines had been animated. Table 1 and Table 2 show the results from the usability questionnaire given during the final user testing session. These results indicated that all users found it easy to understand the coach’s instructions, carry out actions, and move through the program. All users except one found it easy to remember how to do things a second time. Four users found it easy to know what to do in any given situation while 3 found it difficult. This was likely because the coach’s lines had not been fully programmed and a textbox with instructions was used in its place. All users felt immersed in VR, were satisfied with the experience, and enjoyed using the therapy. Two users felt mildly nauseous while using VR while 5 did not.

**Table 1.** First part of usability questionnaire completed by members of the young person’s advisory group (n=7) during Phoenix VR Self-Confidence Therapy testing in August 2022.

Questions	Very easy, n	Easy, n	Difficult, n	Very difficult, n
Knowing what to do in any given VR^a^ situation was…	0	4	3	0
Understanding the coach’s instructions was…	2	5	0	0
Carrying out an action (for example, planting the seeds, throwing the pinecones) was…	2	5	0	0
Moving through the program was…	2	5	0	0
Learning what to do and how to do it was…	1	4	1	1
Remember how to do things a second time was…	4	2	1	0

a VR: virtual reality.

**Table 2.** Second part of usability questionnaire completed by members of the young person’s advisory group (n=7) during Phoenix VR Self-Confidence Therapy testing in August 2022.

Statements	Agree, n	Agree a little, n	Disagree a little, n	Disagree, n
When using the VR^a^, I felt like I was in the situation.	4	3	0	0
I was satisfied with the overall VR experience.	7	0	0	0
I enjoyed using the treatment.	5	2	0	0
The VR made me feel sick.	0	2	0	5

a VR: virtual reality.

### Deliver

This phase of the design process (August 2022-May 2023) included finalizing all activities, debugging, and conducting final usability testing (as part of a cohort study with patients in clinical services). As in the develop phase, an iterative approach was used whereby finalized activities were checked by the research team at the University of Oxford for usability and effectiveness. The script was also finalized during this phase after feedback from users in the cohort study and a last recording with the voice actor for the coach took place in February 2023. The final script is approximately 500 lines. When the design and script were locked in, there was a pure bug-testing phase involving the whole team (clinical psychologists and software developers), during which the application was polished to remove glitches and add final enhancements.

The cohort study for Phoenix examined usability, satisfaction, and initial effectiveness of Phoenix in 12 young people with a diagnosis of psychosis across 3 different mental health trusts in the south of England. Patients were recruited over a 4-month period (December 2022-March 2023). All patients were being seen in National Health Service (NHS) services. The primary outcome was usability (Table 3 and Table 4) and satisfaction with the Phoenix program, with secondary outcomes of positive self-beliefs (measured by the OxPos) and psychological well-being (a full description of this cohort study was provided by Freeman et al [27]). The same usability questionnaire administered during user testing in the develop phase was also used with patients in the case series. Compared with the first usability testing session conducted in the develop phase, all users found it easy to move through the program, understand the coach’s instructions, carry out an action, learn what to do, and remember how to do things a second time. Only one person found it difficult to know what to do in any given situation. This improvement in user experience is likely due to the coach’s lines being fully programmed. Similarly, all users enjoyed the treatment, all but one user were satisfied with the overall VR experience, and all but one user felt immersed in VR when they were using it. Only one user reported that the VR made them feel sick.

**Table 3.** First part of usability questionnaire completed by patients (n=11) in the case series for Phoenix VR Self-Confidence Therapy.

	Very easy, n	Easy,n	Difficult, n	Very difficult, n
Knowing what to do in any given VR^a^ situation was…	7	3	1	0
Understanding the coach’s instructions was…	10	1	0	0
Carrying out an action (for example, planting the seeds, throwing the pinecones) was…	7	4	0	0
Moving through the program was…	7	4	0	0
Learning what to do and how to do it was…	7	4	0	0
Remember how to do things a second time was…	8	3	0	0

a VR: virtual reality.

**Table 4.** Second part of usability questionnaire completed by patients (n=11) in the case series for Phoenix VR Self-Confidence Therapy.

	Agree, n	Agree a little, n	Disagree a little, n	Disagree, n
7. When using the VR^a^, I felt like I was in the situation.	6	4	1	0
8. I was satisfied with the overall VR experience.	9	1	1	0
9. I enjoyed using the treatment.	9	2	0	0
10. The VR made me feel sick.	0	1	1	9

a VR: virtual reality.

## Regulatory Process

As described in the original design brief, Phoenix is certified as a class I medical device in conformity with the essential requirements of Directive 93/42/EEC. The certification process was run in parallel with the design process for Phoenix and involved evaluating risks, software, usability, clinical safety, and efficacy of the application. A medical device consultant provided feedback on how to document the software development to ensure it complied with requirements. This was done via online project management and bug tracking software (Jira/Confluence; Atlassian Corporation). The research team, along with the consultant, developed and completed documentation regarding potential hazards of use and embedded mitigating factors, instructions for use, a data processing impact assessment, software specifications, and a clinical evaluation report (which included data on usability testing, described in the Design Process section).

## Phoenix VR Self-Confidence Therapy

The resulting Phoenix VR Self-Confidence Therapy program is designed to be delivered over the course of approximately 6 weekly sessions. The activities within Phoenix spark positive self-beliefs that are consolidated through real-world activities. Although key positive self-beliefs are embedded within each broad area and explicitly named by the coach, patients are also encouraged to identify additional positive self-beliefs on which to focus. Key techniques, aided by the mental health provider, include helping patients identify their strengths and the activities linked to those strengths, develop the ability to fully immerse themselves in and therefore savor positive activities, and increase engagement in meaningful activities.

Users start out in the welcome room and, after an introduction to Phoenix and tutorial on how to use the program, choose from the 3 environments. They can repeat activities and return to the welcome room at any time. Prior to each activity, they are provided with a description of the purpose and what steps need to be taken to complete it. After completion, Farah provides positive feedback and encouragement. Farah directs users to pay attention to the feelings of enjoyment and achievement elicited by the activities in Phoenix and encourages them to elicit these same feelings in their daily life. Handouts with positive psychology techniques supplement the delivery of Phoenix.

Users also have the option to take the headset home with them to use in between sessions. This means that in-person sessions may or may not involve the use of VR depending on patient preference. The mental health provider helps users set practices to complete between sessions aimed at continuing to build positive self-beliefs, with sessions focused on reviewing these practices and consolidating the learning made from using Phoenix.

## Clinical Evaluation

### Ethical Considerations

Ethical approval for the cohort study and the randomized controlled trial (described below) was obtained from an NHS research ethics committee (22/LO/0273). The study abided by the Ethical Principles of Psychologists and Code of Conduct. The McPin Foundation followed all appropriate safeguarding policies and ethical considerations during the design process.

### Next Steps

Twelve patients completed Phoenix as part of the cohort study and 11 provided outcome data [27]. Uptake of Phoenix was high: 9 users had 6 sessions and 8 patients consistently used Phoenix on their own in between sessions. Satisfaction was high and there were few side effects. All users rated the quality of the VR therapy as good or excellent. An initial evaluation of positive self-beliefs and psychological well-being showed large improvements. Therefore, the cohort study is now being followed up with a randomized controlled trial in 4 mental health trusts in the south of England.

Eighty patients will take part [28]. Half of the participants will receive Phoenix in addition to their usual treatment and half will continue with their usual treatment only. There will be 2 follow-up assessments: one at 6 weeks (after treatment completion) and one at 12 weeks. Assessments will be completed by research assistants who are blind to the treatment allocation. The primary outcome is positive self-beliefs (measured by the OxPos), with secondary outcomes of well-being, everyday confidence, depression, anxiety, hopelessness, activity levels, and quality of life. Service use will also be measured for an analysis of health economics. For those who receive Phoenix, satisfaction and side effects will also be assessed.

## Abbreviations

NHS: National Health Service
OxPos: Oxford Positive Self Scale
VR: virtual reality
VR-CBT: virtual reality–based cognitive behavioral therapy
YPAG: young person’s advisory group
